# A predictive model for prostate cancer incorporating PSA molecular forms and age

**DOI:** 10.1038/s41598-020-58836-4

**Published:** 2020-02-12

**Authors:** Julia Oto, Álvaro Fernández-Pardo, Montserrat Royo, David Hervás, Laura Martos, César D. Vera-Donoso, Manuel Martínez, Mary J. Heeb, Francisco España, Pilar Medina, Silvia Navarro

**Affiliations:** 1 Haemostasis, Thrombosis, Atherosclerosis and Vascular Biology Research Group, Medical Research Institute Hospital La Fe (IIS La Fe), Valencia, 46026 Spain; 2 Data Science. Biostatistics and Bioinformatics Unit. Medical Research Institute Hospital La Fe (IIS La Fe), Valencia, 46026 Spain; 3 Department of Urology, La Fe University and Polytechnic Hospital, Valencia, 46026 Spain; 4 Department of Molecular Medicine, The Scripps Research Institute, La Jolla, CA 92037 USA

**Keywords:** Prostate cancer, Prostate

## Abstract

The diagnostic specificity of prostate specific antigen (PSA) is limited. We aimed to characterize eight anti-PSA monoclonal antibodies (mAbs) to assess the prostate cancer (PCa) diagnostic utility of different PSA molecular forms, total (t) and free (f) PSA and PSA complexed to α_1_-antichymotrypsin (complexed PSA). MAbs were obtained by immunization with PSA and characterized by competition studies, ELISAs and immunoblotting. With them, we developed sensitive and specific ELISAs for these PSA molecular forms and measured them in 301 PCa patients and 764 patients with benign prostate hyperplasia, and analyzed their effectiveness to discriminate both groups using ROC curves. The free-to-total (FPR) and the complexed-to-total PSA (CPR) ratios significantly increased the diagnostic yield of tPSA. Moreover, based on model selection, we constructed a multivariable logistic regression model to predictive PCa that includes tPSA, fPSA, and age as predictors, which reached an optimism-corrected area under the ROC curve (AUC) of 0.86. Our model outperforms the predictive ability of tPSA (AUC 0.71), used in clinical practice. In conclusion, The FPR and CPR showed better diagnostic yield than tPSA. In addition, the PCa predictive model including age, fPSA and complexed PSA, outperformed tPSA detection efficacy. Our model may avoid unnecessary biopsies, preventing harmful side effects and reducing health expenses.

## Introduction

In Europe, prostate cancer (PCa) is the most common solid neoplasm in men, with an incidence rate of 25% of all newly diagnosed cancers and shows the highest death rate after lung and bronchus cancer^1^. Men surviving PCa are the largest population of male cancer survivors and comprise approximately 40% of all. Significant controversy concerning PCa overdetection and overtreatment has led to a search for better markers. Overdetection is a minor problem compared to underdetection. Overtreatment is an ethical problem that could be solved when effective tools to differentiate clinically significant from indolent tumours are adopted. Approximately 1.3 million of prostate biopsies are performed every year in the USA. Most of them are negative but 43% will require a new diagnostic biopsy in 3 years^2^. This situation involves side effects and unnecessary expenses.

Prostate specific antigen (PSA), also known as human glandular kallikrein 3, is a member of the kallikrein family which also includes tissue kallikrein and human glandular kallikrein 2 (hK2). PSA is secreted by prostate epithelial cells^3^ and is present in serum from patients with prostate disease^4^. High levels of PSA are a useful marker for PCa detection^4^, for monitoring follow-up and progression after radical prostatectomy^5^, and for monitoring local or systemic therapy^6,7^, However, levels of PSA are also increased in some patients with benign prostatic hyperplasia, acute prostatitis^8^ or prostate manipulations, leading to unnecessary negative biopsies or to over detection of non-significant cancers. Nonetheless, PSA screening has saved the lives of many men around the world, is an independent variable for PCa, and is a better predictor of cancer than digital rectal examination (DRE) or transrectal ultrasound diagnosis^9^. So, the suggestions to abandon PSA screening are unjustified. Instead, we should refine the diagnosis with better screening and better biopsy performing.

Different new biomarkers, cancer metabolism markers or microRNAs are being investigated to improve diagnosis of PCa in different types of samples, such as serum, urine, semen or cell cultures^10–13^. In addition, different strategies using several PSA molecular forms or ratios, PSA density or velocity^14^, proPSA forms, PSA glycoforms^15^ or PSA in combination with platelet volume and distribution^16^ have been developed in order to improve the specificity of PSA as a biomarker. Moreover, novel score tests to provide the risk of PCa derived from a mathematical algorithm for different kallikrein biomarkers as well as other clinical information, have been developed^17,18^. They are known as: 4 K Score [total PSA (tPSA), free PSA (fPSA) and intact PSA and human kallikrein 2 hK2)]^19,20^, Prostate Health Index (PHI) (tPSA, fPSA ratio and [−2]proPSA)^21,22^ or Stockholm-3 test^23,24^ (tPSA, fPSA and intact PSA, hK2, MSMB, MIC1, genetic polymorphisms, age, family history, previous prostate biopsy, DRE and prostate volume). Another approach, derived from these scores and estimated in large populations, is the risk calculators (RCs) prediction models, developed to assess patient’s individual PCa risk, which show a moderate to well discriminatory ability to predict PCa^25^. There are other prognostic scores based on genetic approaches, but their results are controversial^3,26^. In addition, mutations in kallikrein genes are associated with the risk of PCa and tumour aggressiveness^27,28^.

One of the most promising approaches to improve the specificity of the PSA test to better distinguish between PCa and non-PCa, is the development of assays for measuring different molecular forms of PSA in plasma or serum. PSA is present in circulation in different molecular forms, including fPSA and PSA complexed with α_1_-antichymotrypsin (PSA-α_1_ACT) and with α_2_-macroglobulin (PSA-α_2_M)^29^, although the most relevant forms are fPSA and PSA-α_1_ACT^30,31^. TPSA, fPSA and PSA-α_1_ACT complex levels vary with age, race and ethnicity, body mass index values or the assay kits used^32^. Nevertheless, these variations are mainly associated with prostate disease. The free-to-total (FPR) and complexed-to-total PSA (CPR) ratios have been shown to provide a better discrimination between PCA and non-PCa^33,34^. Additionally, hK2 has about 80% homology to PSA and some anti-PSA antibodies may cross-react with this protein. Therefore, the presence of different molecular forms of PSA and kK2 in serum, illustrates the need to develop new anti-PSA antibodies that do not cross-react with hK2 and may distinguish between fPSA and PSA-α_1_ACT more precisely.

Here, we report the preparation and characterization of eight anti-PSA monoclonal antibodies (mAbs) and their usefulness in specific sandwich ELISAs for tPSA, fPSA and PSA-α_1_ACT complex. We also evaluated the clinical usefulness to discriminate between PCa and non-PCa for these molecular forms, using ROC curves as well as combination of several biomarkers in a predictive model that may enhance sensitivity and specificity of PSA alone. All the assays were performed in citrated plasma samples as we have reported that citrated plasma samples provide higher specificity than serum samples when using FPR and CPR as markers^35^.

## Results

### Characterization of monoclonal anti-PSA antibodies

In our study, eight murine anti-PSA mAbs were generated and characterized: M1, M15, M21, M29, M40, M50, M63, and M73. All of them were of the IgG1 with kappa chain type and none exhibited cross-reactivity with female sera, determined by incubation with different relative concentration of antibodies and PSA (see Supplementary Fig. S1), showing that female serum does not contain any component that competes with PSA for antibody binding. The apparent dissociation constant (Kd) of each mAb for immobilized PSA or PSA in solution is shown in Supplementary Table S1, with the M40 mAb showing the highest affinity.

Only two of our eight mAbs showed cross-reactivity with hK2, especially M73, so it could only be used as a secondary antibody (see Supplementary Fig. S2). Four combinations, M1/M21*, M21/M40*, M40/M50* and M40/M73*, detected fPSA and PSA-α_1_ACT with the same efficiency (see Supplementary Fig. S3). Hence, we selected the pair M40/M73* to set up an ELISA for tPSA. This assay does not recognize hK2, is equimolar (detects all PSA molecular forms in equal molar ratios), and has a detection limit of 0.1 μg/L (see Supplementary Figs. S2–S4).

### Immunoassays for the PSA molecular forms

All combinations with M63 were specific for fPSA (see Supplementary Fig. S4). We selected the pair M63/M50* to set up an assay specific for fPSA. The assay does not detect complexed PSA, has a detection limit of 0.04 μg/L (see Supplementary Fig. S4), and gave no signal with hK2 concentrations up to 20 μg/L.

There were three combinations (M1/M40*, M1/M50* and M1/M73*) that reacted slightly more efficiently with PSA-α_1_ACT than with fPSA (see Supplementary Fig. S4), suggesting that M1 may not be useful to measure PSA-α_1_ACT complex. Therefore, we selected the combination M40/polyclonal anti-α_1_ACT* pair to measure PSA-α_1_ACT in plasma. It gave a good dose-response curve with purified PSA-α_1_ACT diluted both in buffer and in plasma and no signal was obtained either with fPSA up to 2000 μg/L or with purified α_1_ACT up to 500 mg/L. The assay has a detection limit of 0.05 μg/L of complexed PSA (see Supplementary Fig. S4), and gave no signal with hK2 concentrations up to 20 μg/L.

### Clinical usefulness of the PSA molecular forms

We studied 764 patients with benign biopsy (BB) and 301 patients with PCa. Table 1 shows median with the first and third quartiles in brackets, or n with % in parenthesis for tPSA, fPSA, PSA-α_1_ACT, PSA-α_2_M, PSA-hk2, as well as FPR, CPR and FPR/CPR ratio, as markers to discriminate between PCa and BB. The concentration of tPSA, fPSA, PSA-α_1_ACT, PSA-α_2_M, PSA-hK2 and CPR was significantly higher in patients with PCa than in those with BB (P < 0.001, P = 0.012, P < 0.001, P < 0.001 and P < 0.001, respectively), whereas prostate volume, FPR and FPR/CPR ratio were significantly higher in BB than in PCa (*P* < 0.001) (Table 1). Using tPSA levels, we also estimated the PSA density (tPSA/prostate volume) for all patients, which was significantly higher in PCa patients than in BB (*P* < 0.001).

**Table 1:** Clinical characteristics of the cohort of PCa and BB patients studied.

	PCa (n = 301)	BB (n = 764)	*P*-value
Age, years	67 [64–73]	66 [61–70]	<0.001
Prostate volume, cm^3^	34 [26–48]	44 [31–57]	<0.001
PSA density, μg/L*cm^3^	0.23 [0.13–0.45]	0.15 [0.09–0.25]	<0.001
tPSA <4 µg/L	27 (9%)	136 (18%)	<0.001
tPSA ≥4 µg/L	174 (91%)	628 (82%)	
DRE normal	102 (34%)	581 (76%)	<0.001
DRE abnormal	199 (66%)	183 (24%)	
tPSA, µg/L	9.9 [6.2–23.7]	6.5 [4.5–9.4]	<0.001
fPSA, µg/L	1.8 [0.7–4.0]	1.4 [0.8–2.4]	0.012
PSA-α_1_ACT, µg/L	9.1 [5.5–17.4]	4.8 [3.2–6.9]	<0.001
PSA-α_2_M, µg/L	1.9 [0.5–4.4]	1.0 [0.4–1.7]	<0.001
hK2-α_2_M, µg/L	5.2 (2.7–13.6)	3.1 (1.9–5.2)	<0.001
fPSA ratio (FPR)	14 [10–18]	23 [16–32]	<0.001
Complex PSA ratio (CPR)	89 [78–96]	75 [62–86]	<0.001
FPR/CPR ratio	0.16 [0.11–0.23]	0.31 [0.19–0.51]	<0.001
Patients with			
1 biopsy	85 (28%)	384 (50%)	
2 biopsies	134 (45%)	223 (29%)	
3 biopsies	69 (23%)	137 (18%)	
4 biopsies	13 (4%)	20 (3%)	
n (%)			
Patients, n (%):			
- with Gleason score	164 (54.5%)		
- without Gleason score	137 (45.5%)		
*Gleason score 6, n (%)	103 (63%)		
Gleason score ≥7, n (%)	61 (37%)		

Data are presented as median with the first and third quartiles in brackets, or n with % in parenthesis. *Gleason score was also available in 164 PCa patients.

We used two types of analyses to assess the clinical performance of the parameters studied, one through ROC curves grouping patients into subgroups according to their tPSA level, and the other through multivariable logistic regression models with continuous variables, for generalizable results.

Table 2 shows the sensitivity, specificity, and AUC values for the group of patients with tPSA between ≥4 and <10 μg/L. For this subgroup of patients, FPR (AUC = 0.81), CPR (AUC = 0.79) and FPR/CPR ratio (AUC = 0.79) gave a better discrimination than tPSA (AUC = 0.56).

**Table 2:** Sensitivity (%), specificity (%), and AUC for total PSA, free PSA, PSA density, PSA-α_1_ACT, free-to-total PSA ratio (FPR), complexed-to-total PSA ratio (CPR) and FPR/CPR ratio for the 126 patients with PCa and 464 with BB with tPSA between ≥4 and <10 µg/L.

Assay	Cut-off value	Sensitivity	Specificity	AUC (95% CI)
Total PSA, µg/L	>4.04	100	1	0.56 (0.53–0.61)^b^
	>4.20	95	6	
	>4.60	90	14	
	>4.90	85	21	
PSA density	≤0.78	100	2	0.52 (0.43–0.61)
	≤0.55	95	5	
	≤0.49	90	8	
	≤0.41	85	14	
Free PSA, µg/L	≤3.43	100	3	0.73 (0.68–0.76)
	≤2.01	95	25	
	≤1.52	90	45	
	≤1.42	85	49	
PSA-α_1_ACT, µg/L	>2.25	100	2	0.71 (0.67–0.74)
	>3.36	95	18	
	>3.96	90	34	
	>4.05	85	36	
FPR	≤53	100	1	0.81 (0.78–0.84)
	≤27	95	37	
	≤23	90	46	
	≤19	85	61	
CPR	>44	100	3	0.79 (0.75–0.82)
	>69	95	35	
	>72	90	43	
	>79	85	60	
FPR/CPR	≤23	100	1	0.79 (0.76–0.82)
	≤6	95	35	
	≤5	90	50	
	≤4	85	55	

^a^CI, confidence interval; ^b^*P* < 0.001 for the difference in AUC for total PSA *vs* all other parameters. *P* > 0.05 for all other comparisons.

Supplementary Tables S4–S7 show the sensitivity, specificity, and AUC values for the different subgroups analysed: whole cohort of patients, patients with tPSA between ≥2.5 and ≤4 μg/L, patients with tPSA between ≥4 and <10 μg/L and patients with tPSA between 10 and <20 μg/L. For the whole cohort of analysed patients, the FPR/CPR ratio (0.82) and FPR (0.78) gave a better discrimination than tPSA (0.69) (Supplementary Table S4). For the group of patients with tPSA between ≥2.5 and <4 ng/ml, the FPR/CPR ratio (0.77) and FPR (0.76) again gave better discrimination than tPSA (0.53) (Supplementary Table S5). For the tPSA range between ≥10 and <20 μg/L, FPR (0.80) and CPR (0.79) gave the best discrimination compared to tPSA (0.55) (Supplementary Table S7). In this range, using a cut-off point of 4.4 for the FPR/CPR ratio we would have avoided 30% biopsies without losing any PCa patient.

**Figure 1:**
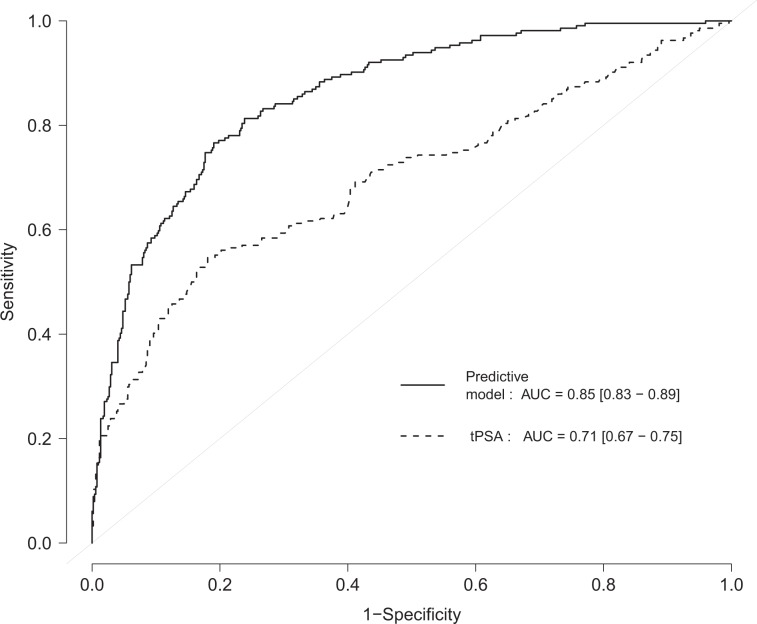
ROC curves for the predictive model (age, tPSA, fPSA and tPSA*fPSA) compared to that obtained for tPSA using mAbs. The area under the curve (AUC) and interquartile range in parenthesis are shown.

Clinical variables, age, PSA density, tPSA, fPSA, PSA-α_1_ACT, PSA-α_2_M, FPR, CPR and FPR/CPR were also analyzed in multivariable logistic regression models. Table 3 shows the different models used for discriminating between PCa and BB using the akaike information criterion (AIC). The best model, according to the AIC criterion, included only the variables: age (OR = 1.75, 95% CI: 1.31–3.36 (per 10 years), *P* < 0.001,), tPSA and fPSA values (OR = 22.55, 95% CI: 13.1–40.5, *P* < 0.001; and OR = 0.05, 95% CI: 0.025–0.095, *P* < 0.001, respectively), as well as their interaction (OR = 1.31, 95% CI: 1.08–1.6, *P* = 0.007) (Table 4). Due to their skewed distribution, tPSA, fPSA and their interaction were log-transformed prior to modelling. With a likelihood ratio test we compared the performance of our elected model and the other models proposed. As depicted in Table 3, our model outperformed all others. Our model substantially improves the predictive capacity of PCa compared to that of tPSA. It achieved an apparent AUC of 0.86 (95% CI: 0.83–0.89) and an optimism-corrected AUC of 0.86, compared to AUC of 0.71 (95% CI: 0.67–0.75) for tPSA (Fig. 1). The formula for predicting the probability (Pr) of PCa would be:

$${\rm{\Pr }}(PCa)=\frac{{e}^{-10.57+0.056\ast Age+3.116\ast \log (tPSA)-2.995\ast \log (fPSA)+0.268\ast \log (tPSA)\ast \log (fPSA)}}{1+{e}^{-10.57+0.056\ast Age+3.116\ast \log (tPSA)-2.995\ast \log (fPSA)+0.268\ast \log (tPSA)\ast \log (fPSA)}}$$

**Table 3:** Different models used for discriminating between PCa and BB patients using the AIC.

Model	AIC	LR-test *P*-value 1^st^ *vs*. others
Age + log(tPSA) + log(fPSA) + log(tPSA)*log(fPSA)	629.61	—
Age + log(tPSA) + log(fPSA)	635.88	0.004
Age + CPR + FPR	704.86	<0.001
Age + log(PSA-α_1_ACT) + log(fPSA) + log(tPSA) + log(PSA-α_2_M)	637.12	<0.001
Age + log(PSA-α_1_ACT) + log(fPSA) + log(tPSA) + CPR + FPR + log(PSA-α_2_M)	631.34	0.91

The model with the lower AIC value was selected as the best model. *Indicates an interaction relationship.

**Table 4:** Multivariable logistic regression models constructed to analyze the probability of PCa occurrence using clinical variables and different combinations of PSA molecular forms.

	OR	Lower 95%	Upper 95%	P-value
Age	1.058	1.027	1.09	<0.001
log(tPSA)	22.554	13.122	40.451	<0.001
log(fPSA)	0.05	0.025	0.095	<0.001
log (tPSA)*log (fPSA)	1.308	1.084	1.6	0.007

Only those variables that estimate the best akaike information criterion *(*AIC) were shown. *Indicates an interaction relationship.

In order to assess whether our selected model was really better than by biopsy all, we performed a decision curve analysis comparing our selected model to total PSA and biopsy all (Supplementary Fig. S5). The results show that our model improves the standardized net benefit over all the range of thresholds compared to biopsy all and over most of the threshold values compared to total PSA values. Thus, standardized net benefit values are higher in our model compared to biopsy all starting at a probability of 4%. This difference is statistically significant starting at a probability of 13%.

In order to ease the interpretation of the results of the model, we represented its sensitivity and specificity profile plot (Supplementary Fig. S6). Using this profile plot we may select a sensitivity (for example 90%), obtaining a specificity of 58%.

We also generated an effect plot depicting the relationship between tPSA, fPSA and the probability of cancer (Supplementary Fig. S7). It shows that increasing levels of tPSA are associated with higher probabilities of cancer, and increasing levels of fPSA are associated with lower probabilities of cancer. The effect of one biomarker can mask the effect of the other at extreme values (i.e., low values of tPSA always result in low probabilities of cancer no matter what the fPSA values are, and high values of fPSA always result in low probabilities of cancer no matter how high the tPSA values are). We also provided as supplementary material a spreadsheet (see supplementary Dataset) for performing straightforward predictions. For example, for an 80 years old patient, if tPSA is 9 ng/mL and fPSA is 1.5 ng/mL, the calculated risk of PCa is 44.5% (see supplementary Dataset).

Furthermore, we estimated the improvement of our new predictive model versus the classical markers (tPSA levels) using the net reclassification improvement (NRI) and the integrated discrimination improvement (IDI). We observed that our predictive model had an NRI value of 0.870, splitted as NRI for the event (PCa) of 0.374 and NRI for the non-event (BB) of 0.496 (Supplementary Table S7). The IDI value was 0.201. So, the NRI and IDI scores indicate that our combination of several biomarkers in a predictive model may enhance sensitivities [increase for PCa (sensitivity) = 0.142] and specificities [decrease for BB (specificity) = 0.0587] of PSA alone, improving the ability to estimate the risk of PCa.

**Figure 2:**
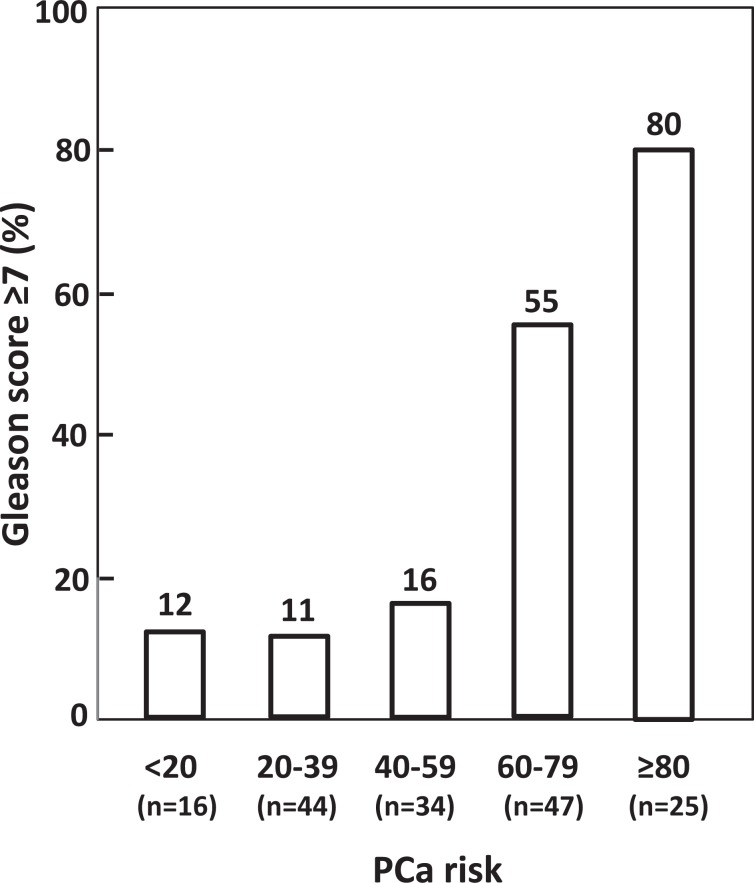
Percentage of PCa patients with Gleason score ≥7 in relation to PCa risk.

Additionally, we have analyzed the correlation between the Gleason score and the risk of PCa, calculated with our predictive model. For these analyses involving the Gleason score we only included samples from patients with reliable Gleason scores. In many cases, we couldn’t find the data, or the grading was not reliable because of the sample specimen analyzed. In the 164 PCa patients for whom we had the Gleason score, the coefficient of correlation was r = 0.56 (95% CI: 0.45–0.66, P < 0.0001) and the number of patients with tumours with Gleason scores ≥ 7 increased as the PCa risk increased (Fig. 2). Moreover, the AUC estimated by introducing the PCa risk, calculated with our model, and the dichotomized Gleason score (<7 and ≥ 7), was 0.83 (95% CI: 0.77–0.89, P < 0.0001). Using a cut-off value of PCa risk > 50%, the specificity was 39% for a sensitivity of 95%. This means that, using a sensitivity of 95%, we could have avoided about 39% of biopsies to PCa patients with a Gleason score <7.

## Discussion

Over the past decade, PSA has been shown to be the most valuable diagnostic and prognostic marker in oncology. However, its reliability as screening tool for PCa remains controversial due to its lack of specificity. Several non-malignant conditions of the prostate, such as BB, are associated with an increased PSA levels^35^. Alternative biomarkers for PCa have emerged with the aim of increasing the diagnostic specificity, prognosis and staging of this cancer^3,14,36–41^. Some of these new diagnostic tools are related to PSA, such as PHI^21,40^, 4 K Score^19,20^ and the STHLM3 test^23,24^, or RCs to assess the PCa risk of a patient^25^. However, their clinical applicability is controversial and remain a matter of personal choice whether to use it in daily clinical practice.

PSA circulates in two forms, fPSA (30%) or PSA complexed to α_1_ACT (70%). The FPR or the CPR have become important markers to improve the specificity of tPSA and the differential diagnosis of BB and PCa^33,42–45^. There are different immunoassays for detection of these PSA molecular forms with different assay manufacturers. In systematic review, Roddam *et al*.^34^ described the diagnostic ability of the FPR and CPR in men with tPSA levels between 2 and 10 ng/mL, and its impact on clinical practice. They concluded that their use in this segment of patients could reduce the number of unnecessary biopsies whilst maintaining a high cancer detection rate.

Accordingly, we proposed the use of specific homemade ELISAs for tPSA, fPSA and PSA-α_1_ACT complex by using different pairs of antibodies that do not recognize hK2. These assays are equimolar and show detection limits and variation coefficients that are in all cases adequate, and have been validated in a large cohort of patients with PCa and BB (more information is shown in Supplementary material).

Our results show that PSA-α_1_ACT, FPR, CPR and FPR/CPR ratio significantly increase the diagnostic yield of tPSA when patients are classified according to different tPSA ranges (see Supplementary Tables S4–S6). In the diagnostic gray zone of 4 to 10 μg/l tPSA, using a cut-off value of 27 for FPR, 69 for CPR or 6 for FPR/CPR ratio (95% specificity), we could avoid about 35% of biopsies compared to 6% of biopsies avoided when using a cut-off value of 4.2 μg/l tPSA.

Our results compare well with those obtained with the PHI and [−2]proPSA markers, showing similar or better clinical performance for PCa detection^46^. There, the PHI was significantly higher in PCa patients than in patients without PCa, with an AUC of 0.70. Our results show AUCs for FPR, CPR and FPR/CPR, ranging from 0.79 to 0.81.

In order to improve the discrimination between PCa and BB, we compared our new predictive model to the classical marker (tPSA levels) using the NRI and the IDI. Our results, compared to those obtained with the PHI, with an AUC ranging from 0.70 to 0.77^45,46^, showed that our predictive model including age, tPSA, fPSA and the interaction of tPSA and fPSA, rendered a superior AUC (0.85; 95% CI = 0.83–0.89), demonstrating a better clinical performance for PCa detection. Additionally, our model shows another advantage, the independence from prostate volume. Only age is required as a valuable clinical factor, combined with the PSA molecular forms. Similar results are found when comparing our predictive model with the results described for the 4 K Score panel^20^, which could distinguish PCa and BB with good accuracy, with an AUC from 0.81 to 0.84, respectively, similar to that estimated with our predictive model (0.85). When we compared our predictive model with the seven well known RCs, that shown an AUC range between 0.64 and 0.72, we observed again how our predictive model exhibits a better discriminatory ability to predict PCa. And if we select only patients with clinically significant PCa, the RCs study with the highest AUC is 0.77, lower than the AUC obtained in our predictive model for all patients^25^.

Finally, Vickers *et al*.^19^, described a predictive model similar to ours, based on how additional kallikreins (fPSA, intact PSA, and hK2) could enhance discrimination of PCa diagnosis compared to a classical laboratory model (including age and tPSA) or a classical clinical model (including age, tPSA and DRE). They described a homemade ELISA to identify intact PSA and hK2 kallilkreins, based on modified mAbs with less nonspecific assay interference. This study obtained an AUC of 0.64 for tPSA levels, 0.70 including DRE, and 0.76 including age and additional kallikreins. We propose an alternative mAb design to quantify other markers (fPSA and PSA-α_1_ACT), based on equimolar tests that show no unspecific interactions with others kallikreins. Our strategy has allowed us to develop a predictive model with highest AUC, simple and easy to introduce in daily clinical practice.

Although our study had as main objective to identify a predictive model of PCa, given the current interest in identifying patients with more aggressive tumours, we decided to also analyze whether the predictive model of PCa obtained was also able to discriminate between aggressive and indolent phenotypes of PCa, for which we included in the study the 164 PCa patients for whom we had a reliable Gleason score. We observed a significant correlation between the Gleason score and PCa risk obtained with our model. Furthermore, the number of patients with a score ≥7 increased with the increase in the risk of PCa (Fig. 2), and the risk of PCa was significantly higher in patients with Gleason score ≥7 tumours (70%) compared with Gleason score 6 tumours (39%) (*P* < 0.0001). A similar result was reported by Stephan *et al*.^45^ using the PHI index. From the AUC performed introducing the PCa risk, calculated with our model, and the dichotomized Gleason score (<7 and ≥7), using a cut-off point of PCa risk >50%, the specificity was 39% for a sensitivity of 95%, indicating that with our model we could have avoided about 39% of biopsies to PCa patients with a Gleason score <7.

A limitation of this study is that our predictive model needs to be validated in a prospective study before prostate biopsy has been conducted, and needs an external validation using a multicenter study population. However, prospective population-based screening strategies are difficult to implement in daily clinical practice and require approval by local authorities. Another limitation is the unfeasibility to gather the variables needed for the estimation of proPSA and 4K-panel, so no head-to-head comparison of the different methods could be made. Finally, of the 301 patients with PCa we only had 164 patients with the available Gleason score. It would be necessary to confirm our results with a higher number of PCa patients.

In conclusion, we have developed and validated specific and sensitive immunoassays to quantify different PSA molecular forms. Our results using ROC curves for different tPSA ranges show that the FPR and CPR significantly increase the discriminating power of tPSA, fPSA and PSA-α_1_ACT.

In addition, the combination of tPSA, fPSA and age in a predictive model, increases the diagnostic power of tPSA, widely used in clinical practice, and may identify patients with a more aggressive tumours. Thus, the use of our predictive model may avoid unnecessary biopsies while high sensitivity is maintained, thus reducing unnecessary side effects in hundreds of thousands of patients every year and consequently unnecessary health expenses. A diagnosis in time and in early stages is very advisable and valuable, since it represents the critical stage regarding treatment and survival of the patient.

## Material and Methods

### Study subjects

This case-finding study included 1,065 patients with at least one prostate biopsy, 764 with BB and 301 with PCa, selected for having a positive DRE and/or a tPSA ≥4 μg/L. Samples were collected between 1997 and 2001.

PCa was objectively diagnosed by transrectal ultrasound-guided prostate biopsy, or by examination of tissue specimens following transurethral prostatectomy (incidental diagnosis). According to clinical situation, only 17 of the 301 PCa patients studied (5.7%) underwent transurethral resection for prostatic obstruction, a small percentage that would not cause deviation in the study population.

A diagnosis of BB was considered when patients had a negative biopsy and also when patients had an increase in prostate volume (>40 cm^3^) along with the following symptoms: obstructive symptoms, hesitant or intermittent micturition, decreased strength and thinning of the urinary stream gauge.

All participants were enrolled after giving written informed consent according to protocols approved by the ethics review board at La Fe University Hospital (reference no. 3009/0085). The procedures followed were in accordance with the Helsinki Declaration of 1975 as revised in 2008.

### Blood collection

Blood was collected into Vacuette© sodium citrate tubes, centrifuged at 1,800 x g for 30 minutes at 4 °C and plasma samples were aliquoted and frozen at −70 °C until analysis.

### Reagents

Purified hK2 and a monoclonal anti-hK2 antibody (HK1G86.1) that does not cross-react with PSA were provided by Hybritech Inc. (San Diego, CA). Biotin-NHS, human α_1_ACT and α_2_M, and rabbit anti-human α_2_M (IgG fraction) were obtained from Calbiochem (La Jolla, CA). Rabbit anti-human α_1_ACT (IgG fraction) was purchased from Dako A/S (Glostrup, Denmark). Aprotinin-Sepharose, casein, bovine serum albumin, horseradish peroxidase (HRP, type VI, RZ = 3.2), anti-mouse IgG (goat)-HRP, anti-mouse IgG (rabbit)-HRP whole molecule, Tween 20, hydrogen peroxide (H_2_O_2_), O-phenylenediamine (OPD), benzamidine chloride, dimethyl sulfoxide and dithiothreitol were from Sigma Chem. Co. (St. Louis, MO). Glutaraldehyde and 1,10-phenanthrolinium chloride were from E. Merck (Darmstadt, Germany). Streptavidin-HRP (SAHRP), biotinylated horse polyclonal anti-mouse IgG antibody and NBT/BCIP were from Pierce (Thermo Scientific, Waltham, MA USA). Streptavidin-alkaline phosphatase was from Bio-Rad. CM-Sephadex, Sephacryl S-200 and CNBr-activated Sepharose 4B were obtained from Pharmacia (Uppsala, Sweden).

### Production and characterization of monoclonal anti-PSA antibodies

See supplementary results for the production, purification, isotyping, calculation of the apparent Kd (Supplementary Table S1), biotinylation, serum competition (Supplementary Fig. S1), SDS-PAGE and Western blots, reactivity of anti-PSA mAbs on immunoblots towards fPSA and PSA-α_1_ACT complex (Supplementary Table S2), competition between unlabelled and labelled mAbs for binding to PSA (Supplementary Table S3), cross-reactivity with hK2 (Supplementary Fig. S2) and reactivity of several pairs of anti-PSA mAbs towards fPSA and PSA-α_1_ACT complex (Supplementary Fig. S3).

### Immunoassay for total PSA

The assay for tPSA was performed with M40 as capturing antibody and biotinylated (*) M73 as detecting antibody. Plates were coated with 5 mg/L of M40. After washing and blocking with blocking buffer, 50 μL/well of duplicated samples or calibrators were added and incubated for 1 h at room temperature (RT). After washing, 50 μL/well of M73* at a dilution of 1/8000 in 0.01 mol/L Tris-HCl, pH 7.4, 0.14 mol/L NaCl, 0.5 g Thimerosal per liter, 0.5 mL Tween 20 per liter, was added and incubated for 1 h at RT. After washing, SAHRP at 1/4000 was applied as described above. Colour was developed with OPD substrate and the reaction was stopped after 5 min with 35 μL/well of 4 mol/L H_2_SO_4_.

### Immunoassay for PSA -α_1_ACT complex

The assay for PSA-α_1_ACT was performed with M40 as capturing antibody and a polyclonal HRP-labelled anti-α_1_ACT antibody as detecting antibody. Plates were coated with 5 mg/L of M40. After washing and blocking, 50 μL/well of duplicated samples or calibrators were added and incubated for 1 h at RT. After washing, 50 μL/well of HRP-labelled anti-α_1_ACT antibody at a dilution of 1/2000 was added and incubated for 1 h at RT. After washing, colour was developed with OPD substrate and the reaction was stopped after 5 min with 35 μL/well of 4 mol/L H_2_SO_4_.

### Immunoassay for fPSA

The assay for fPSA was performed with M63 as capturing antibody and M50* as detecting antibody. Plates were coated with 5 mg/L of M63. After washing and blocking, 50 μL/well of duplicated samples or calibrators were added and incubated for 1 h at RT. After washing, 50 μL/well of M50* at a dilution of 1/10000 was added and incubated for 1 h at RT. After washing, SAHRP at 1/4000 was applied as described above. Colour was developed with OPD substrate and the reaction was stopped after 5 min with 35 μL/well of 4 mol/L H_2_SO_4_.

### Immunoassay for PSA -α_2_M complex

The assay for PSA-α_2_M complex was performed as reported before for the activated protein C-α_2_M complex^47^. Briefly, plasma samples were pre-treated with dithiothreitol and then with iodoacetamide, in order to expose PSA epitopes. The M40 anti-PSA antibody was used as coating antibody and biotinylated anti-IgG antibody was used as detecting antibody. The detection range of the PSA-α_2_M assay was 0.1 to 4.0 ng/ml of complex.

### Statistical analysis

Data were summarized using median, standard deviation and 1^st^ and 3^rd^ quartile in the case of continuous variables and relative and absolute frequencies in the case of categorical variables. Multivariable logistic regression models were constructed with different combinations of the PSA molecular forms and clinical variables. Selection of the best model for discriminating between PCa and BB was performed using the AIC^48^. Calibration of the final model was assessed using a scatter plot of the predicted versus observed probabilities using 500 bootstrap replicates of the sample. Although the bootstrap is not a substitute of a large sample external validation, it is the best method for assessing calibration when no external validation is feasible. The predictive ability of the model was assessed by estimating an optimism-corrected area under the curve (AUC) for the receiver operator characteristic (ROC) analysis, and using 1000 bootstrap replicates. All these statistical analyses were performed using R (version 3.4.2) and R-packages pROC (1.10.0) and rms (5.1–1). Moreover, the improvement of the new (multivariable logistic regression model) versus the classical marker, tPSA, were estimated by using the NRI and the IDI. For that, the R-package Hmisc (4.1–1) was used for the NRI and IDI of logistic regression models^49^. NRI and IDI values were provided as a complement of the AUC values for the different models, following specific guidelines^49^, and providing only 95% CI for NRI and IDI. The AUC to assess the diagnostic accuracy of each parameter was calculated using a commercially available computer program for medical statistics^50^. Analysis of ROC curves using clinical groups was performed on patients according to tPSA levels: a) tPSA between 0.76 and 975 µg/L (whole cohort), b) ≥ 2.5 and <4.0 μg/L, c) ≥ 4 and <10 μg/L and d) ≥ 10 and <20 μg/L. Using the same statistical program, we estimated the AUC introducing the PCa risk, calculated with our model, and the dichotomized Gleason score (<7 and ≥7).

## Supplementary information

Supplementary information.

Supplementary information2
