# Supplementary material for: A predictive model for prostate cancer incorporating PSA molecular forms and age
Source: Sci Rep. 2020 Feb 12;10:2463. doi: 10.1038/s41598-020-58836-4 (PMC7016114; doi:10.1038/s41598-020-58836-4)
Supplement: Supplementary file 1 — Supplementary information. [file 41598_2020_58836_MOESM1_ESM.docx]

**Supporting Information**

**A predictive model for prostate cancer incorporating PSA molecular forms and age**

Julia Oto^1†^, Álvaro Fernández-Pardo^1†^, Montserrat Royo^1^, David Hervás^2^, Laura Martos^1^, César D. Vera-Donoso^3^, Manuel Martínez^3^, Mary J. Heeb^4^, Francisco España^1^_,_ Pilar Medina^1*^, Silvia Navarro^1*^

†These authors contributed equally to this work

^1^Haemostasis, Thrombosis, Atherosclerosis and Vascular Biology Research Group, La Fe Medical Research Institute.

^2^Bioscience Unit. La Fe Medical Research Institute. La Fe University and Polytechnic Hospital, Valencia, Spain.

^3^Department of Urology. La Fe University and Polytechnic Hospital, Valencia, Spain.

^4^Department of Molecular Medicine, The Scripps Research Institute, La Jolla, CA, USA.

**Correspondence:**

Silvia Navarro

IIS La Fe-Hospital Universitario y Politécnico La Fe

Torre A, 5ª Planta, Lab. 5-18

Av. Fernando Abril Martorell 106

46026 Valencia, Spain

Phone: 34-961246640

E-mail: navarro_silros@gva.es

or

Pilar Medina

IIS La Fe-Hospital Universitario y Politécnico La Fe

Torre A, 5ª Planta, Lab. 5-09

Av. Fernando Abril Martorell 106

46026 Valencia, Spain

Phone: 34-961246636

E-mail: medina_pil@gva.es

**Supplementary Material & Methods**

# Purification of PSA

PSA was purified from seminal fluid as described by Sensabaugh and Blake^1^ with the following modifications: each semen donation was collected into 4 mL of a solution containing 200 mmol/L benzamidine-HCl and 20 mmol/L phenanthroline-HCl in distilled water, to reduce PSA inactivation. Semen was immediately centrifuged at 10,000g for 5 min at 4 ºC and the supernatant was stored at -80 ºC. Samples from 14 donors were thawed at 37 ºC for 5 min, mixed with 2 volumes of 0.01 mol/L potassium phosphate buffer, pH 6.9, containing 30 mmol/L benzamidine-HCl and 10 mmol/L phenanthroline-HCl, and dialyzed against 20 volumes of the same buffer. The dialyzed material was centrifuged at 5,000g for 15 min and applied to a CM-Sephadex column and then to a Sephacryl S-200 column. Fractions containing PSA from the last column were concentrated and dialyzed on a PM-10 Amicon membrane against 0.025 mol/L Tris-HCl, pH 7.4, 0.5 mol/L NaCl, 0.2g Na-azide per liter, and applied to an aprotinin-Sepharose column as previously described^2^. The final preparation had a concentration of 2.5 g/L as determined by absorption at 280 nm, using an extinction coefficient ε^0.1%^ of 1.84. A concentration of 2.70 and 2.53 g/L was calculated using the Hybritech Tandem-E assay and a homemade PSA assay,^3^ respectively.

# Preparation of polyclonal anti-PSA antibodies

# Specific rabbit anti-human PSA antibody and anti-PSA IgG labelled with HRP were obtained following standard procedures as previously reported.^4^

# Preparation of PSA-α_1_ACT complex

# The complex was partially purified from plasma of a patient with 86% of the PSA complexed to α_1_ACT using a reported method^5^ with slight modifications. The plasma was filtered on a 2.6x90 cm Sephacryl S-200 column equilibrated in 0.1 mol/L potassium phosphate buffer pH 6.9, 0.5 mol/L NaCl, 0.2g Na-azide per liter, at 14 mL/h, collecting fractions of 1 mL. The degree of separation between PSA and PSA-α_1_ACT complex was analyzed by specific ELISAs for total PSA (tPSA) and PSA-α_1_ACT complex. The filtration effectively separated most of the free PSA (fPSA) (30 kDa) from PSA-α_1_ACT complex (90 kDa). No efforts were made to separate free α_1_ACT from PSA-α_1_ACT complex, since it is known that an excess of inhibitor stabilizes the complex. Fractions containing PSA-α_1_ACT were pooled and concentrated on a PM-10 Amicon membrane. The final preparation contained a tPSA concentration of 206.6 μg/L, and a concentration of PSA complexed with α_1_ACT of 191.3 μg/L (7% of the tPSA was in the fPSA form).

# Production and purification of monoclonal antibodies

# Hybridomas secreting anti-PSA monoclonal antibodies (mAbs) were obtained by immunization of BALB/c mice with 60 μg PSA purified from human seminal plasma by standard procedures as previously described.^6^ Hybridomas were screened in ELISA plate wells containing immobilized PSA. To this end, plates were coated at 4 ºC overnight with 50 μL PSA (3 mg/L) in 0.1 mol/L sodium carbonate buffer, pH 9.6. After washing and blocking with 0.01 mol/L Tris‑HCl, 0.14 mol/L NaCl, pH 7.4 (TBS) buffer containing 10 g casein per liter, 0.2 ml Tween 20 per liter and 0.4 g sodium azide per liter (blocking buffer), undiluted supernatants were incubated with immobilized PSA for 2 h at room temperature (RT). mAbs were detected with an anti-mouse IgG (goat)-HRP. mAbs where purified from mouse ascites fluid using a column of PSA coupled to CNBr-activated Sepharose 4B according to the manufacturer’s specifications.

# Antibody isotyping

# Isotypes of mAbs were determined using the Mouse Typer Sub-Isotyping Kit from Bio-Rad.

# Determination of the apparent dissociation constants

# The apparent dissociation constants (Kd) were calculated by direct ELISA. Wells were coated with 100 g/L of purified PSA overnight at 4 ºC. Then, several concentrations of mAbs (ranging from 0.003 nmol/L to 600 nmol/L) in blocking buffer were incubated with immobilized PSA for 1 h at RT. The mAb bound was detected with anti-mouse IgG (rabbit)-HRP whole molecule. Furthermore, we evaluated the affinity of each mAb for PSA in solution. Plates were coated overnight at 4 ºC with mAbs at 5 mg/L. After washing and blocking, PSA was added at different concentrations (ranging from 0.098 to 400 μg/L) in blocking buffer and plates were incubated for 1 h at RT. Bound PSA was detected with a polyclonal HRP-labelled anti-PSA antibody.

# The Kd was calculated from the concentration of mAb (or purified PSA) that gave an absorbance equal to 50% of the absorbance obtained with a saturated concentration of antibody (or PSA).

# Biotinylation of antibodies

# mAbs and anti-IgG antibody were labelled with biotin-NHS-ester, according to the manufacturer’s specifications. Briefly, 1 mL (2 g/L) of each antibody was incubated for 2 h at room temperature with 0.2 mL (1.5 g/L) of biotin-NHS dissolved in dimethyl sulfoxide. After dialysis, immunoreactivity of biotinylated mAbs were assessed by ELISA with PSA immobilized directly on plates. Plates were coated with 50 μL/well of 100 μg/L PSA in 0.1 mol/L sodium carbonate buffer, pH 9.6, at 4 ºC overnight. After blocking and washing, the native and biotinylated mAbs were incubated with immobilized PSA at different concentrations in blocking buffer (ranged from 0.025 to 6 mg/L). After washing with 0.01 mol/L Tris-HCl, pH 7.4, 0.14 mol/L NaCl, 0.5 g Thimerosal per liter, 0.5 mL Tween 20 per liter (conjugated buffer), plates were incubated with 50 μL/well of anti-mouse IgG (rabbit)-HRP whole molecule at a 1/1000 dilution. Colour was developed with the OPD substrate during 8 min and the reaction was stopped with 4 mol/L H_2_SO_4_.

# Serum competition

# Serum from women was diluted 1/2 with 0.01 mol/L sodium phosphate, 0.137 mol/L NaCl, 2.7 mmol/L KCl, pH 7.4 and 50 μl/well was added to ELISA plates, previously coated with 50 μL/well of 0.5 or 5.0 mg/L anti-PSA mAbs. After blocking and washing, 50 μL of 180 μg/L PSA (wells with 0.5 mg/L mAb) or either 90 μg/L or 500 μg/L PSA (wells with 5 mg/L mAb) was added and incubated for 2 h at RT. Bound PSA was detected with a polyclonal HRP-labelled anti-PSA antibody at a concentration of 6 ng/L. Finally, colour was developed by adding 50 µL of a solution of 400 mg/L OPD in 0.1 mol/L Na citrate, 0.2 mol/L Na phosphate, pH 5.0, containing 400 μL of 30% H_2_O_2_ per liter. The reaction was stopped after 5 min by adding 35 μL/well of 4 mol/L H_2_SO_4_. The absorbance of the colour formed was read in an ELISA microplate autoreader at 492 nm.

# SDS-PAGE and Western blots

# SDS-PAGE was performed as reported by Laemmli et al^7^. PSA or PSA-α_1_ACT was denatured under non-reducing or reducing (10 mmol/L dithiothreitol) conditions. After electrophoresis on 10% SDS-polyacrylamide gels, proteins were transferred to nitrocellulose membranes. The membranes were blocked with 5% non-fat milk in TBS buffer for 2 h at RT. mAbs where diluted to their optimal concentrations in 1% non-fat milk in TBS buffer and incubated with membranes 2 h at RT. Membranes were washed 3 times for 10 min and incubated with biotinylated-anti mouse IgG at 0.75 mg/L for 40 min. Thereafter, membranes where washed 3 times for 10 min and incubated with streptavidin-alkaline phosphatase for 20 min at RT. After washing, colour was developed with NBT/BCIP.

# Sandwich ELISA assays

# The eight mAbs were tested in all possible combinations in a sandwich format, excepting M15 because of its low affinity. Plates were coated overnight at 4 ºC with 50 μL/well of 5 mg/L mAb. After washing and blocking, PSA or PSA complexed to α_1_ACT was added at different concentrations (ranging from 15 μg/L to 0.029 μg/L) in blocking buffer and plates were incubated for 1 h at RT. Thereafter, plates were incubated for 1 h at RT with 50 μl/well of 2 mg/L biotinylated anti-PSA mAb in 0.01 mol/L Tris-HCl, pH 7.4, 0.14 mol/L NaCl, 0.5 g thimerosal per liter, 0.5 mL Tween 20 per liter. After washing, plates were incubated for 20 min at RT with 50 μL/well of SAHRP diluted at 1/5000 in 0.01 mol/L Tris-HCl, pH 7.4, 0.14 mol/L NaCl, 0.5 g Thimerosal per liter, 0.5 mL Tween 20 per liter. After washing, colour was developed by adding 50 μL/well of a solution of 400 mg/L OPD in 0.12 mol/L Na citrate, 0.2 mol/L Na phosphate, pH 5.0, containing 400 μL of 30% H_2_O_2_ per liter. The reaction was stopped by adding 35 μL/well of 4 mol/L H_2_SO_4_. The absorbance of the colour formed was read in an ELISA microplate autoreader at 492 nm.

# Cross-reactivity with hK2

# To elucidate whether the anti-PSA mAbs recognized hK2 we performed sandwich ELISAs. Plates were coated overnight at 4 ºC with 50 μl of 5 mg/L mAb. After washing and blocking, 50 μL of hK2 at 20 mg/L or PSA at 20 μg/L were added and incubated for 1 h at RT. For comparison, a specific anti-hK2 mAb was also analyzed. HK2 or PSA bound were detected with the polyclonal anti-PSA labelled with HRP.

# Competitive binding to immobilized PSA

# Additional investigation into epitope recognition by the eight mAbs was facilitated by competitive binding to immobilized PSA. PSA antigen was immobilized on microtiter wells, and the ability of any antibody to block the binding of another was evaluated. The ability to block binding would indicate identical or overlapping epitope specificity. Plates were coated with 50 μL/well of 100 mg/L fPSA. After blocking and washing, the plates were incubated for 1 h with one of the mAbs at 50 mg/L in blocking buffer or only with blocking buffer. Serial dilutions of the biotinylated forms of the antibodies (ranging from 0.25 to 10 ng/L) were then loaded into to the plate and incubated for 1 h. After washing the plates, SAHRP was loaded as described above. Colour was developed with OPD substrate and the reaction was stopped with 4 mol/L H_2_SO_4_ as described above. Competition was recorded as positive when the absorbance decreased below 75% of that observed without unlabeled antibody.

# PSA calibrators

# Serial dilutions of four different PSA calibrators were used in the ELISAs for tPSA, fPSA or PSA-α_1_ACT complex. One calibrator was fPSA purified as indicated above, and its concentration was calculated from the absorption at 280 nm, using an extinction coefficient ɛ^0.1%^ of 1.84. The second calibrator was partially purified PSA-α_1_ACT prepared as indicated above. The other two calibrator were obtained from the National Institute for Biological Standards and Control (NIBSC); one was the prostate-specific antigen (free) first international standard (NIBSC code 96/668), which consists of fPSA, and the other was the prostate-specific antigen (90:10) first international standard (NIBSC code 96/670) [PSA (90/10) Standard], which consists of a mixture of 90% PSA bound to α_1_ACT and 10% fPSA. To better compare the results, for PSA-α_1_ACT complex assay we only considered the 90% of PSA that is in the complexed form in the 90:10 first international standard, whereas for fPSA assay we only considered the 10% that is in the free form.

***Supplementary Results***

**Characterization of monoclonal antibodies**

Eight anti-PSA mAbs (M1, M15, M21, M29, M40, M50, M63, and M73) that gave a high signal in the screening test from two hybridoma fusions were selected. All of them were of the IgG_1_ with kappa chain type and none exhibited cross-reactivity with female sera, assessed by incubation with different relative concentration of antibodies and PSA (see supplementary Figure S1), showing that female serum does not contain any component that competes with PSA for antibody binding.

The apparent dissociation constant (K_d_) of each mAb for immobilized PSA or PSA in solution is shown in supplementary Table S1. The affinity of each antibody varied depending on whether PSA was or was not immobilized. The greatest affinity for immobilized PSA corresponded to M40 antibody (0.3 x 10^–9^ mol/L), whereas the M15 mAb showed the lowest affinity (178.4 x 10^-9^ mol/L). For PSA in solution, the M29 and M40 mAbs showed the highest affinity (8.9 x 10^-9^ mol/L and 10.1 x 10^-9^ mol/L, respectively) whereas the M15 gave the lowest affinity (>200 x 10^-9^ mol/L). As seen in supplementary Table S1, the K_d_**´**s are more homogeneous for PSA in solution than for immobilized PSA. In general, all antibodies showed more affinity for immobilized PSA than for PSA in solution, except M50. This suggests that M50 recognizes an epitope that could be altered by slight conformational changes that may occur when PSA is bound to the ELISA plate.

SDS-PAGE and immunoblotting studies were performed to characterize the nature of the epitopes recognized by the mAbs (see supplementary Table S2). The results suggested that M1, M21, M29 and M73 mAbs detected linear epitopes on the PSA molecule that remained intact on the reduced forms of both fPSA and PSA-α_1_ACT complex. In contrast, four mAbs, M15, M40, M50 and M63, seemed to recognize conformation-dependent epitopes on PSA-α_1_ACT complex that are lost under reducing conditions.

All pair combinations of the eight mAbs (excepting M15) were tested for reactivity with fPSA and PSA-α_1_ACT in ELISAs. Some examples of sandwiching behaviour of the combinations are shown in supplementary Figure S3. We found that some pairs (all with M63) didn’t detect PSA-α_1_ACT, for example, the M63/M50* and M63/M40* combinations seem to be specific for fPSA. Other pairs (M1/M21*, M21/M40*, M40/M50*, M40/M73* and M73/M50*) recognized fPSA and PSA-α_1_ACT with the same efficiency, and could be used to measure tPSA. Only three combinations (M1/M40*, M1/M50* and M1/M73*) reacted a bit more efficiently with PSA-α_1_ACT than with fPSA. An * above indicates a biotinylated detecting antibody.

The eight mAbs were examined for the capacity to inhibit the binding of each labelled counterpart to PSA. Supplementary Table S3 shows the results obtained. Competition was recorded when the absorbance decreased below 75% of that observed without unlabeled antibody. Results revealed two main clusters of epitopes, one recognized by M21, M29 and M63, and the other by M1, M15 and M40. The antibodies in each group seemed to recognize an identical epitope/s topographically closely related. The epitope for M73 did not appear to overlap with those of any of the other antibodies.

Supplementary Figure S2 shows the cross-reactivity with hK2 of the different anti-PSA mAbs. Only M73 significantly cross-reacted with purified hK2 in solution, compared to a specific anti-hK2 mAb.

**Calibration and limit detection of the immunoassays for the PSA molecular forms**

It is essential that methods for PSA are equimolar in their response to fPSA and PSA-α_1_ACT and calibrated to the International Standards to minimize the likelihood of clinical errors due to calibration issues^8^.

Supplementary Figure S4, top panel, shows calibration curves for the tPSA assay. Calibration curves of purified PSA diluted in blocking buffer were linear within the range of 0.3 to 10 μg/L under the assay conditions used. No differences were seen when the dilutions of PSA were made in control plasma (data not shown). Serial dilutions of two reference PSA preparations obtained from the National Institute for Biological Standards and Control (NIBSC, UK) containing different proportion of free and complexed PSA gave similar responses than those obtained with purified PSA used in the assay. The detection limit of the assay, defined as the concentration of PSA that gave an absorbance equal to that of the assay buffer + 3 SD (calculated from 15 replicates), was 0.1 μg/L of PSA. The intra- and interassay CVs for tPSA were 3-14% and 5-11%, respectively, at PSA concentrations of 0.22-2.6 μg/L (n=10).

Supplementary Figure S4, central panel, shows calibration curves for the PSA-α_1_ACT assay. Calibration curves of partially purified PSA-α_1_ACT diluted in blocking buffer were linear within the range of 0.25 to 9 μg/L under the assay conditions used. No differences were seen when the dilutions of the complex were made in control plasma. Serial dilutions of PSA (90:10) Standard gave similar responses to those obtained with partially purified complex in the assay, whereas the PSA (free) Standard gave no signal in the assay up to a PSA concentration of 200 μg/L. To make clearer the comparison in the signals, only 90% of PSA that is in the complexed form in the PSA (90:10) Standard was considered. The detection limit of the assay, defined as the concentration of PSA complexed to α_1_ACT that gave an absorbance equal to that of the assay buffer + 3 SD (calculated from 15 replicates), was 0.05 μg/L of PSA. The intra- and interassay CVs were 2-7% and 6-9%, respectively, at PSA concentrations of 0.2-2.2 μg/L (n=10).

Supplementary Figure S4, lower panel, shows calibration curves for the fPSA assay. Calibration curves using the PSA (free) Standard diluted in blocking buffer were linear within the range of 0.05 to 2 μg/L under the assay conditions used. No differences were seen when the dilutions of PSA (free) Standard were made in control plasma. Serial dilutions of the PSA (90:10) Standard gave parallel responses to PSA (free) Standard in the assay. Again, only the 10% of PSA that is in the free form in the PSA (90:10) Standard was considered. The detection limit of the assay, defined as the concentration of PSA that gave an absorbance equal to that of the assay buffer + 3 SD (calculated from 15 replicates), was 0.04 μg/L PSA. The intra- and interassay CVs for tPSA were 4-11% and 5-15%, respectively, at PSA concentrations of 0.06-1.00 μg/L (n=10).

We used ROC curves to assess the clinical performance of the assays. Supplementary Tables S4, S5 and S6 show the sensitivity, specificity, and AUC of each assay in the 3 subgroups analyzed according to the tPSA. For the whole cohort of patients analyzed (tPSA between 0.76 and 975 μg/L), the FPR/CPR ratio (0.82) and the FPR (0.78) gave higher discrimination than tPSA (0.69) (supplementary Table S4). For the subgroup of patients with tPSA between ≥2.5 and <4 μg/L, the FPR/CPR (0.77) and the FPR (0.76) again had the best discriminating potency compared to tPSA (0.53) (supplementary Table S5). For the tPSA range between ≥4 and <10 μg/L, the FPR (0.81), CPR (0.79) and the FPR/CPR ratio gave better discrimination than tPSA (0.56) (supplementary Table S6). Finally, for the tPSA range between ≥10 and <20 μg/L, all parameters analyzed showed better discrimination than tPSA (supplementary Table S7). In this range, using a cutoff point of 4.4 for the FPR/CPR ratio we would have avoided 30% biopsies without losing any PCa patient.

Supplementary Figure S5 shows a decision curve analysis comparing the Standardized Net Benefit among our developed model (new model), total PSA values (PSA) and biopsy for all patients (All).

Supplementary Figure S6 shows the sensitivity and specificity profile plot for the predictive model.

Supplementary Figure S7 shows an effect plot depicting the relationship between tPSA and fPSA values and the probability of PCa.

We also estimated the improvement of our new predictive model versus the classical markers (tPSA levels) using the net reclassification improvement (NRI) and the integrated discrimination improvement (IDI) (Supplementary Table S8).

Supplementary Dataset (excel file) allows performing straightforward PCa predictions, according to our predictive model.

***Supplementary References***

**1.** Sensabaugh, G.F. & Blake, E.T. Seminal plasma protein p30: simplified purification and evidence for identity with prostate specific antigen. *J. Urol.* **144**, 1523-1526 (1990).

**2.** Christensson, A, Laurell, C.B. & Lilja, H. Enzymatic activity of prostate-specific antigen and its reactions with extracellular serine proteinase inhibitors. *Eur. J. Biochem.* **194**, 755-763 (1990).

**3.** España, F. *et al*. Prostate-specific antigen and its complexes with alpha 1-antichymotrypsin in the plasma of patients with prostatic disease. *Eur. Urol.* **30**, 512-518 (1996).

**4.** España, F. & Griffin, J.H. Determination of functional and antigenic protein C inhibitor and its complexes with activated protein C in plasma by ELISA's. *Thromb. Res*. **55**: 671-682 (1989).

**5.** Christensson, A. *et al*. Serum prostate specific antigen complexed to alpha 1-antichymotrypsin as an indicator of prostate cancer. *J. Urol*. **150**, 100-105 (1993).

**6**. España, F., Estellés, A., Griffin, J.H. & Aznar, J. Interaction of plasma kallikrein with protein C inhibitor in purified mixtures and in plasma. *Thromb. Haemost.* **65**: 46-51 (1991).

**7.** Laemmli, U.K. Cleavage of structural proteins during the assembly of the head of bacteriophage T4. *Nature*. **227**: 680-685 (1970).

**8.** Roddam, A.W., Rimmer, J., Nickerson, C. & Ward, A.M. Programme NPCRM. Prostate-specific antigen: bias and molarity of commercial assays for PSA in use in England. *Ann. Clin. Biochem*. **43**(**Pt 1**): 35-48 (2006).

# *Supplementary Tables & Figures*

**Supplementary Table S1. Apparent dissociation constants (K_d_) of monoclonal anti-PSA antibodies for PSA.**

mAb Apparent K_d_ (nM)

(immobilized PSA) (PSA in solution)

M1 26.0 154.7

M15 178.4 >200

M21 0.6 17.1

M29 4.2 8.9

M40 0.3 10.1

M50 112.9 27.7

M63 8.7 11.5

M73 2.4 12.9

**Supplementary Table S2. Reactivity of anti-PSA monoclonal antibodies on immunoblots towards free PSA and PSA-α_1_ACT complex.** *Linear and conformation-dependent epitope categories are based on antibody reactivity with free PSA and PSA-α_1_ACT complex in reducing and nonreducing SDS-polyacrylamide gels. +++, strong staining; ++, moderate staining; +, weak staining; -, no staining; F, antibody showed reactivity to intact PSA and several PSA fragments.

Nonreducing SDS-PAGE Reducing SDS-PAGE

Antibody free PSA PSA-α_1_ACT free PSA PSA-α_1_ACT

#### Linear epitopes*

#### M1 +++ +++ +++F +++

#### M21 +++ +++ ++ ++

#### M29 +++ ++ ++ +

#### M73 +++ +++ ++ ++

#### Conformational-dependent epitopes

#### M15 + ++ + -

#### M40 +++ ++ ++ -

#### M50 +++ ++ ++F -

#### M63 +++ + + -

**Supplementary Table S3. Competition between unlabelled and labelled monoclonal antibodies for binding to PSA.** C, competition. N, no competition. Left, unlabeled antibody. Top, labelled antibody.

|  | M21 | M29 | M63 | M1 | M15 | M40 | M50 | M73 |
| --- | --- | --- | --- | --- | --- | --- | --- | --- |
| M21 | C |  |  |  |  |  |  |  |
| M29 | C | C |  |  |  |  |  |  |
| M63 | C | C | C |  |  |  |  |  |
| M1 | N | N | N | C |  |  |  |  |
| M15 | N | N | N | C | C |  |  |  |
| M40 | N | C | N | C | C | C |  |  |
| M50 | N | N | N | N | N | C | C |  |
| M73 | N | N | N | N | N | N | N | C |

**Supplementary Table S4. Sensitivity (%), specificity (%), and AUC for total PSA, free PSA, PSA density, PSA-α_1_ACT, free-to-total PSA ratio (FPR), complexed-to-total PSA ratio (CPR) and FPR/CPR ratio for the 301 patients with PCa and 764 with benign biopsy, with tPSA between 0.76 and 975 µg/L (whole cohort).** ^a^ CI, confidence interval; *^b^ P*<0.001 for the difference in AUC for: a) tPSA *vs* fPSA, tPSA *vs* fPSA, tPSA *vs* PSA-α_1_ACT and tPSA *vs* FRP/CPR ratio; b) fPSA vs PSA-α_1_ACT, fPSA vs FPR, fPSA vs CPR and fPSA vs PSA-α_1_ACT; c) PSA-α_1_ACT *vs* CPR and PSA-α_1_ACT *vs* FPR/CPR ratio; d) FPR *vs* CPR and FPR *vs* FPR/CPR ratio; e) CPR *vs* FPR/CPR ratio.

**Assay Cut-off point Sensitivity Specificity AUC (95% CI)***

Total PSA, µg/L >0.78 100 0 0.69 (0.67-0.72)b

>3.23 95 13

>4.15 90 20

>5.00 85 31

PSA density ≤0.89 100 2.2 0.51 (0.43-0.58)

≤0.56 95 7.2

≤0.49 90 9.4

≤0.33 85 18

Free PSA, µg/L ≥0.08 100 0 0.54 (0.51-0.57)

>0.39 95 4

>0.55 90 11

>0.63 85 14

PSA-α_1_ACT, µg/L >0.68 100 1 0.76 (0.73-0.79)

>2.67 95 15

>3.60 90 34

>4.13 85 41

FPR ≤71 100 1 0.78 (0.75-0.80)

≤30 95 28

≤24 90 46

≤21 85 56

CPR >20 100 0 0.74 (0.64-0.81)

>57 95 16

>61 90 24

>66 85 33

FPR/CPR ≤23 100 5 0.82 (0.80-0.84)

≤6.47 95 35

≤4.80 90 49

≤4.18 85 56

**Supplementary Table S5. Sensitivity (%), specificity (%), and AUC for total PSA, PSA density, free PSA, PSA-α_1_ACT, free-to-total PSA ratio (FPR), complexed-to-total PSA ratio (CPR) and FPR/CPR ratio for the 20 patients with PCa and 87 with benign biopsy, with tPSA between ≥2.5 and <4 µg/L.** ^a^ CI, confidence interval; *^b^ P*<0.001 for the difference in AUC for tPSA *vs* all other parameters. *P*>0.05 for all other comparison.

**Assay Cut-off point Sensitivity Specificity AUC (95% CI)***

Total PSA, µg/L ≥2.50 100 0 0.53 (0.44-0.63)^b^

>2.81 95 21

>2.97 90 31

>2.98 85 33

PSA density ≤0.81 100 3 0.62 (0.34-0.85)

≤0.62 95 7

≤0.53 90 11

≤0.47 85 15

Free PSA, µg/L ≤0.91 100 25 0.74 (0.64-0.63)

≤0.63 95 57

≤0.62 90 58

≤0.61 85 58

PSA-α_1_ACT, µg/L >1.53 100 6 0.70 (0.60-0.78)

>1.98 95 18

>2.16 90 31

>2.26 85 33

FPR ≤31 100 17 0.76 (0.66-0.83)

≤28 95 22

≤21 90 54

≤18 85 61

CPR >63 100 16 0.74 (0.64-0.81)

>67 95 18

>79 90 49

>81 85 50

FPR/CPR ≤15 100 14 0.77 (0.68-0.85)

≤13 95 35

≤4.80 90 49

≤4.18 85 56

**Supplementary Table S6. Sensitivity (%), specificity (%), and AUC for total PSA, free PSA, PSA density, PSA-α_1_ACT, free-to-total PSA ratio (FPR), complexed-to-total PSA ratio (CPR) and FPR/CPR ratio for the 126 patients with PCa and 464 with benign biopsy, with tPSA between ≥4 and <10 µg/L.** ^a^ CI, confidence interval; ^b^ *P*<0.001 for the difference in AUC for total PSA *vs* all other parameters. *P*>0.05 for all other comparison.

**Assay Cut-off point Sensitivity Specificity AUC (95% CI)**

Total PSA, µg/L >4.04 100 1 0.56 (0.53-0.61)^b^

>4.20 95 6

>4.60 90 14

>4.90 85 21

PSA density ≤0.78 100 2 0.52 (0.43-0.61)

≤0.55 95 5

≤0.49 90 8

≤0.41 85 14

Free PSA, µg/L ≤3.43 100 3 0.73 (0.68-0.76)

≤2.01 95 25

≤1.52 90 45

≤1.42 85 49

PSA-α_1_ACT, µg/L >2.25 100 2 0.71 (0.67-0.74)

>3.36 95 18

>3.96 90 34

>4.05 85 36

FPR ≤53 100 1 0.81 (0.78-0.84)

≤27 95 37

≤23 90 46

≤19 85 61

CPR >44 100 3 0.79 (0.75-0.82)

>69 95 35

>72 90 43

>79 85 60

FPR/CPR ≤23 100 1 0.79 (0.76-0.82)

≤6 95 35

≤5 90 50

≤4 85 55

**Supplementary Table S7. Sensitivity (%), specificity (%), and AUC for total PSA, PSA density, free PSA, PSA-α_1_ACT, free-to-total PSA ratio (FPR), complexed-to-total PSA ratio (CPR) and FPR/CPR ratio for the 66 patients with PCa and 130 with benign biopsy, with tPSA between ≥10 and <20 µg/L.** ^a^ CI, confidence interval; *^b^ P*<0.001 for the difference in AUC for total PSA *vs* all other parameters. *P*>0.05 for all other comparison.

**Assay Cut point Sensitivity Specificity AUC(95% CI)^a^**

Total PSA, µg/L >10.12 100 4 0.55 (0.48-0.62)^b^

>10.20 95 5

>10.76 90 15

>11.00 85 19

PSA density ≤0.27 100 8 0.62 (0.34-0.85)

≤0.25 95 16

≤0.22 90 21

≤0.18 85 25

Free PSA, µg/L ≤6.71 100 6 0.78 (0.71-0.84)

≤3.33 95 25

≤3.14 90 50

≤2.90 85 56

PSA-α_1_ACT, µg/L >6.68 100 18 0.77 (0.70-0.82)

>3.36 95 38

>3.96 90 44

>4.05 85 56

FPR ≤38 100 19 0.80 (0.74-0.86)

≤30 95 33

≤23 90 59

≤20 85 65

CPR >44 100 3 0.79 (0.75-0.82)

>69 95 35

>72 90 43

>79 85 60

FPR/CPR ≤4.4 100 30 0.78 (0.64-0.89)

≤4.3 95 31

≤4.2 90 32

≤2.6 85 67

**Supplementary Table S8. Net Reclassification Improvement (NRI) and Integrated Discrimination Improvement (IDI) to determine the improvement of the new predictive model (multivariable logistic regression model) *versus* total PSA levels to estimate the risk of PCa.** *Benign biopsy.

|  | Predictive model *vs* PSA (95% CI) |
| --- | --- |
| NRI | 0.870 (0.725-1.015) |
| NRI for PCa | 0.374 (0.250-0.498) |
| NRI for BB* | 0.496 (0.422-0.571) |
| IDI | 0.201 (0.171-0.232) |
| Increase for PCa (sensitivity) 0.142 | |
| Decrease for BB (specificity) 0.059 | |

**Supplementary Figure S1. Serum competition with purified PSA for the eight immobilized monoclonal anti-PSA antibody binding.** Serum from women (+) or buffer (-) was diluted to 50% with 0.137 mol/L NaCl, 2.7 mmol/L KCl, pH 7.4 and incubated in microtiter plates coated with 0.5 or 5.0 mg/L anti-PSA mAb and then with 0.18 mg/L PSA (wells with 0.5 mg/L mAb) or either 0.09 mg/L or 0.5 mg/L PSA (wells with 5 mg/L mAb), as indicated in Methods. (A) 0.5 mg/L mAb and 0.18 mg/L PSA. (B) 5 mg/L mAb and 0.09 mg/L PSA. (C) 5 mg/L mAb and 0.5 mg/L PSA.


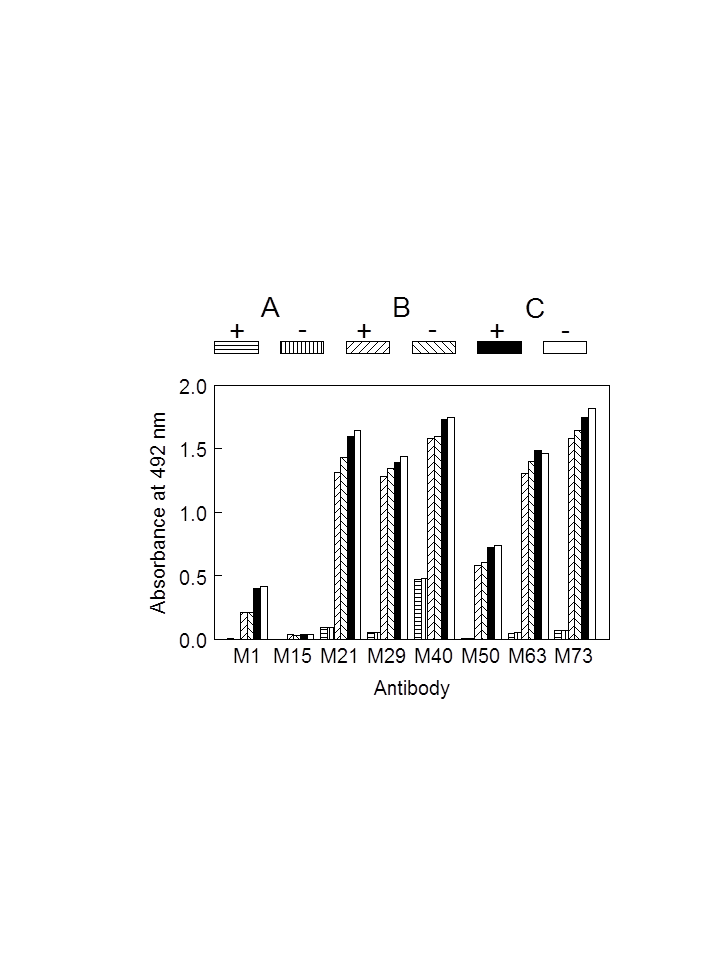


**Supplementary Figure S2. Cross-reactivity of monoclonal anti-PSA antibodies with purified hK2.** ELISA plates coated with the corresponding mAb were incubated with 20 μg/L hK2 or 20 μg/L PSA. For comparison, a specific anti-hK2 mAb was also analyzed. hK2 or PSA bound was detected with polyclonal anti-PSA labelled with HRP.


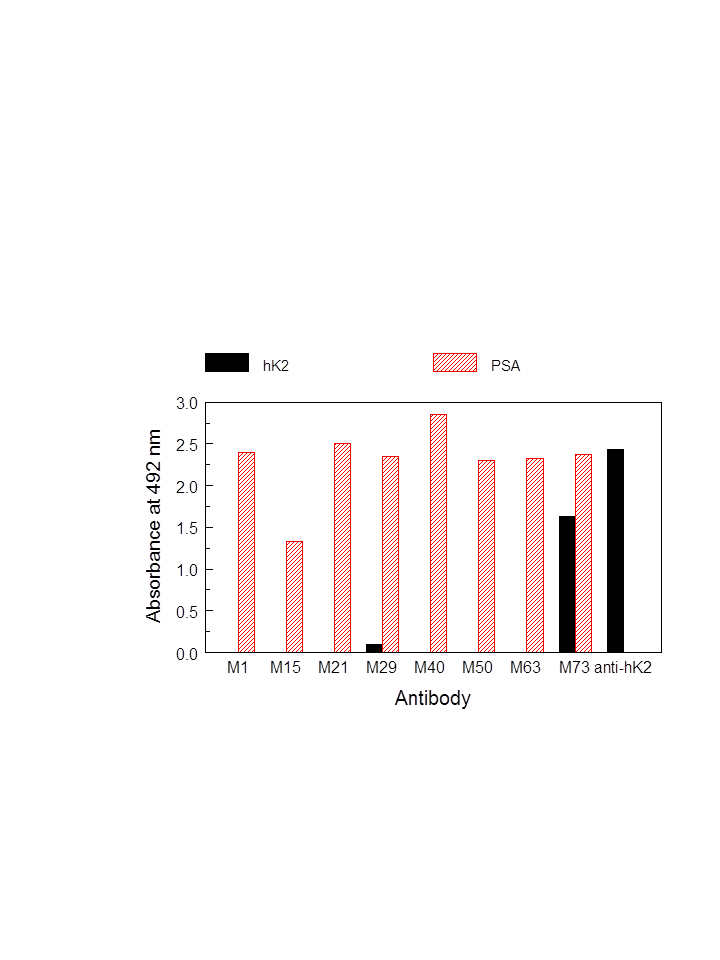


**Supplementary Figure S3. Reactivity of several pairs of anti-PSA mAbs towards free PSA and PSA:α_1_ACT.** ELISA plates, coated with the indicated unlabeled antibody, were incubated with free PSA (●) or PSA:α_1_ACT complex (▲) at different concentrations as indicated. PSA bound was detected with the corresponding labelled (*) antibody. PSA:α_1_ACT is expressed as µg/L of PSA in complex with α_1_ACT.

PSA, μg/L

**Supplementary Figure S4. Calibration curves for total PSA, PSA-α_1_ACT complex, and free PSA immunoassays.** For PSA-α_1_ACT, PSA is expressed as μg/L of PSA in complex with α_1_ACT. The following capture and detecting mAb were used: For total PSA, M40 and M73*; for PSA-α_1_ACT complex, M40 and rabbit anti-human α_1_ACT*; for free PSA, M63 and M50*, respectively. The asterisk indicates labelled antibody.


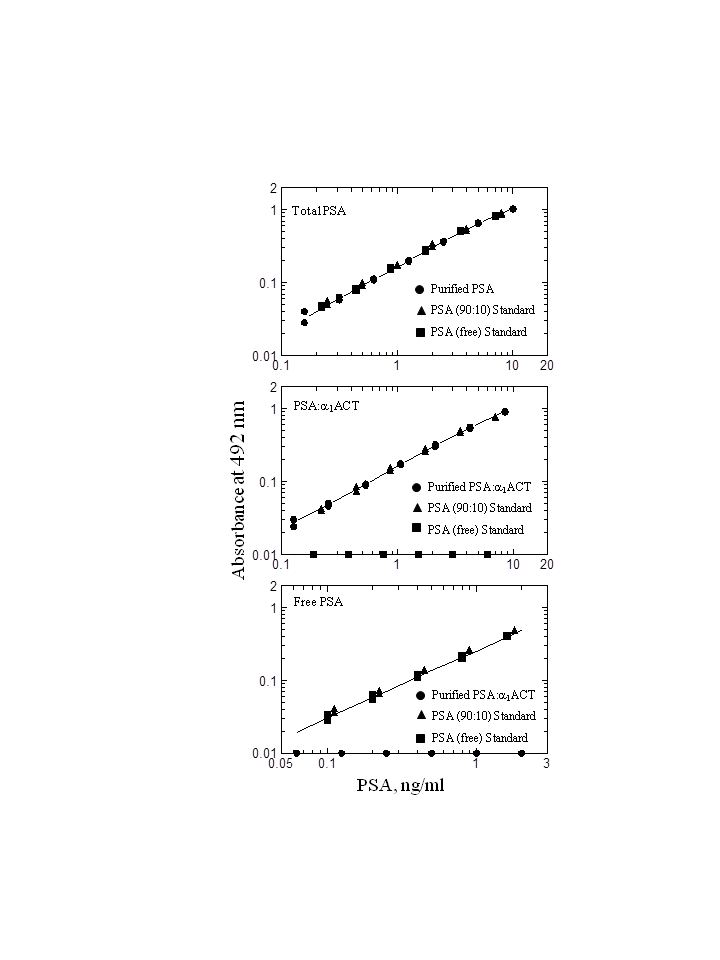


Absorbance at 492nm

PSA, μg/L

**Supplementary Figure S5. Decision curve analysis comparing the Standardized Net Benefit among our developed model (new model), total PSA values (PSA) and biopsy for all patients (All).**

**
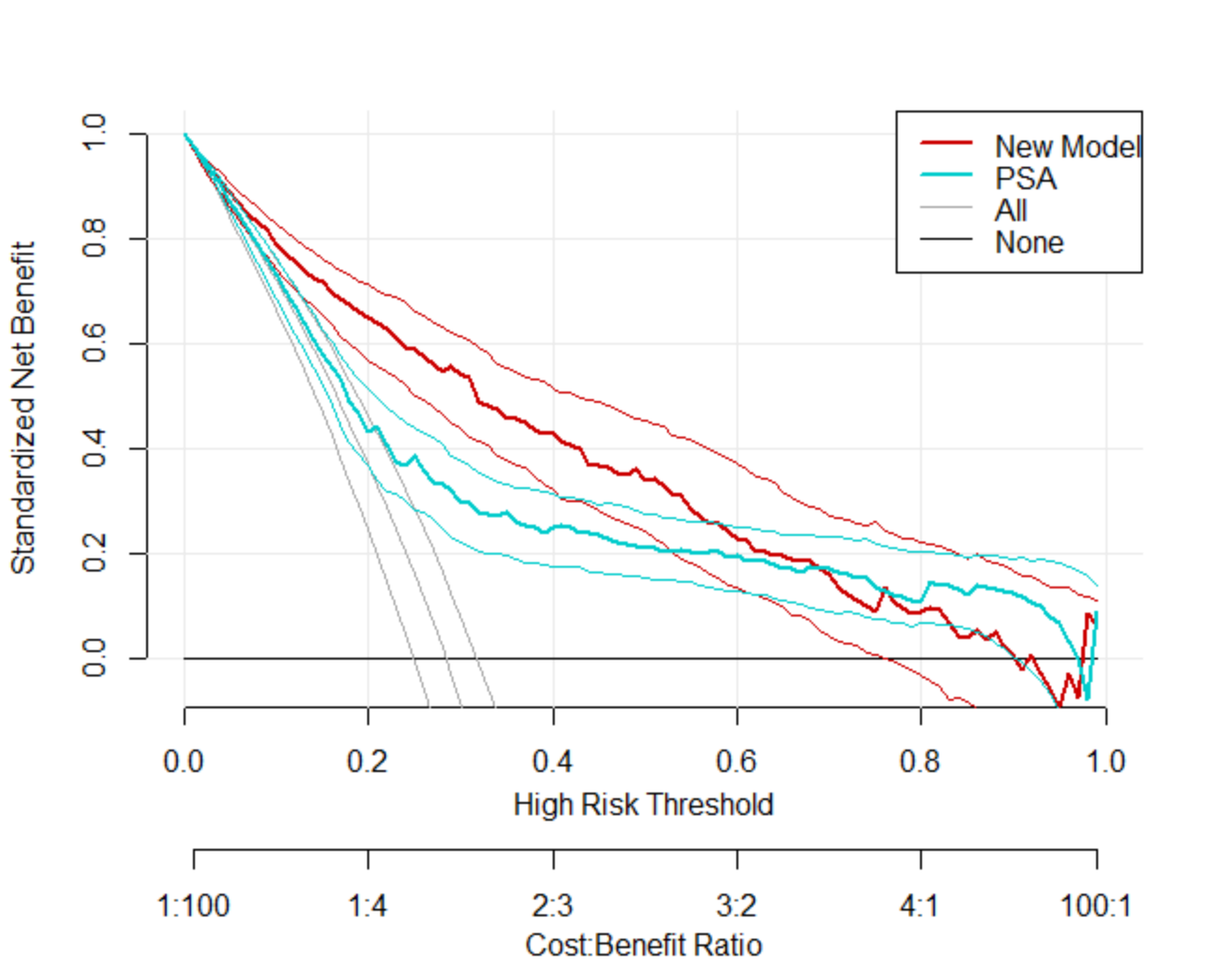
**

**Supplementary Figure 6. Sensitivity and specificity profile plot for the predictive model.**

**Supplementary Figure S7.** **Effects plot depicting the relationship between total PSA (tPSA) and free PSA (fPSA)** **values and the probability of cancer.** Red values represent higher PCa probabilities and blue values represent lower PCa probabilities.
